# Seven-year changes in the epidemiology of ESKAPE pathogens and their antimicrobial resistance in intensive care units of a tertiary hospital: a retrospective observational study

**DOI:** 10.3389/fmed.2026.1995724

**Published:** 2026-10-27

**Authors:** Mirko Mihalj, Ana Milas, Ivan Keser, Svjetlana Grgić, Sanja Jakovac, Božo Šušak

**Affiliations:** 1 Clinic for Anesthesiology, Reanimatology, Intensive Care Medicine and Pain Therapy, University Clinical Hospital Mostar, Mostar, Bosnia and Herzegovina; 2 School of Medicine, University of Mostar, Mostar, Bosnia and Herzegovina; 3 Department for Anesthesia and Critical Care, General Hospital “Prim. Dr. Abdulah Nakaš”, Sarajevo, Bosnia and Herzegovina; 4 Clinic for Infectious Diseases, University Clinical Hospital Mostar, Mostar, Bosnia and Herzegovina; 5 Department for Microbiology and Molecular Diagnostics, University Clinical Hospital Mostar, Mostar, Bosnia and Herzegovina

**Keywords:** antibiotic resistance, epidemiology, ESKAPE pathogen, hospital-acquired infections, intensive care unit

## Abstract

**Background:**

ESKAPE pathogens are the leading cause of hospital-acquired infections (HAIs) in intensive care units (ICUs) and are increasingly associated with multidrug resistance, posing a major challenge to patient management worldwide. This study evaluated seven-year changes in the epidemiology of ESKAPE pathogens and their antimicrobial resistance in ICUs of a tertiary hospital.

**Methods:**

This retrospective observational surveillance study analysed microbiological isolates obtained from patients with hospital-acquired infections treated in the multidisciplinary ICU in 2017 and in the subsequently formed surgical and non-surgical ICUs in 2024 at least 48 h after admission to the ICU. The distribution of ESKAPE pathogens and antimicrobial susceptibility patterns were compared between study periods and ICU settings using *χ*^2^ or Fisher's exact test, with statistical significance set at *p* < 0.05.

**Results:**

A total of 1,113 microbiological isolates were analysed. ESKAPE pathogens accounted for more than 70% of microbiologically confirmed hospital-acquired infections in all investigated ICUs. Although their overall proportion remained stable over the seven-year period, the distribution of individual pathogens changed substantially. Acinetobacter baumannii remained the predominant pathogen in the multidisciplinary and non-surgical ICUs, whereas Klebsiella pneumoniae became the leading isolate in the surgical ICU. Antimicrobial resistance increased significantly, particularly among A. baumannii and K. pneumoniae. In 2024, carbapenem resistance in K. pneumoniae approached 75%, while A. baumannii exhibited resistance exceeding 75% to most routinely tested antimicrobial agents, leaving very limited therapeutic options.

**Conclusions:**

Although the overall prevalence of ESKAPE pathogens remained relatively unchanged, significant shifts occurred in the distribution of individual pathogens together with a substantial increase in antimicrobial resistance over the seven-year period. Continuous local microbiological surveillance is essential for optimizing empirical antimicrobial therapy, supporting antimicrobial stewardship programs and strengthening infection prevention strategies in intensive care units.

## 1 Introduction

Antimicrobial resistance (AMR) is the ability of microorganisms to become increasingly resistant to an antimicrobial drug to which they were previously susceptible. This results in infections persisting in the body and increasing the risk of spreading to the human and animal population through food, water, and the environment (1). A recent study claimed that in 2021, there were 1.14 million deaths attributable to bacterial AMR and 4.71 million worldwide deaths associated with bacterial AMR (2). The same study estimated that in 2050, there will be 1.91 million annual deaths attributable to AMR globally and 8.22 million annual deaths associated with AMR, with regions like South Asia, Southeast Asia, East Asia, Oceania, and sub-Saharan Africa experiencing the highest predictive burden of AMR (2).

Antibiotic resistance is more pronounced in hospital settings, especially in Intensive Care Units where invasive procedures such as mechanical ventilation, tracheostomy, and catheterization are frequently performed, increasing rates of nosocomial infections. In addition, the prolonged and frequent use of antibiotics in ICUs is one of the most significant factors contributing to the higher prevalence of multidrug-resistant microorganisms in this setting (3). Also, large prevalence surveys have shown that the highest prevalence of nosocomial infections is in patients admitted to intensive care units (4).

The “ESKAPE pathogens” is an acronym introduced to denote a group of nosocomial pathogens with a high capability to develop antibiotic resistance and thus “escape” the effects of different antibiotics (5). The acronym ESKAPE includes *Enterococcus faecium*, *Staphylococcus aureus*, *Klebsiella pneumoniae*, *Acinetobacter baumannii*, *Pseudomonas aeruginosa*, and *Enterobacter species*, which are responsible for most hospital-acquired infections (5). Due to their high antimicrobial resistance, the World Health Organization (WHO) classified the ESKAPE pathogens as priority pathogens requiring the development of new antibiotics (6). Among them, based on disease impact and the availability of effective medications, *Acinetobacter baumannii* and *Enterobacterales* have been designated as critical priority pathogens, while the others are categorized within the high-priority group (6).

The aim of this study was to evaluate changes in ESKAPE pathogens' epidemiology and antimicrobial resistance (AMR) in the ICUs of University Hospital Mostar, Bosnia and Herzegovina, during two study periods: January 2017 to December 2017 and January 2024 to December 2024.

## 2 Materials and methods

We conducted a retrospective, single-center, observational surveillance study that analyzed microbiological isolates obtained from patients with hospital-acquired infections admitted to the intensive care units of the University Clinical Hospital Mostar during two predefined study periods (2017 and 2024). This study includes data from patients with treated in a multidisciplinary ICU (MICU) involving surgical (general surgery, trauma patients, etc) and non-surgical patients (patients with respiratory insufficiency of various causes, post-resuscitation patients, patients with infectious diseases, metabolic disorders, etc.) from January 1, 2017, to December 31, 2017 (7). Since then, owing to an increased patient volume, the original multidisciplinary ICU has been reformed into a separate surgical ICU (SICU) for general surgery and trauma patients, and a new non-surgical ICU (NSICU) was formed for non-surgical patients requiring ICU treatment (patients with respiratory insufficiency of various causes, post-resuscitation patients, patients with infectious diseases, metabolic disorders, etc.). This study also included data from patients with nosocomial infections treated in those two ICUs (SICU and NSICU) from January 1, 2024, to December 31, 2024. These ICUs are part of the Clinic for Anesthesiology, Reanimatology, Intensive Care Medicine and Pain Therapy at the University Clinical Hospital Mostar. The 2017 MICU dataset has been reported previously regarding the epidemiology of hospital-acquired infections (5). In the present study, these data were re-analyzed within a new study framework and compared with newly collected data from the surgical and non-surgical ICUs in 2024, with a particular focus on temporal changes in pathogen distribution and antimicrobial resistance.

Samples with negative cultures or Candida spp. isolates were excluded from the analysis.

All procedures followed were in accordance with the ethical standards laid down in the 1964 Declaration of Helsinki and its later amendments. Because this was a retrospective database analysis, informed consent was not required, and since no patients' data were collected or used, Ethics Committee approval was deemed unnecessary.

The data used were collected from the electronic medical records and analyzed. The study included microbiological isolates obtained from patients at least 48 h after admission to the ICU. Antimicrobial resistance was determined from isolated causative agents in sputum samples from non-intubated patients, endotracheal aspirates from intubated or tracheotomy patients, and blood and urine samples. Pathogen identification and antimicrobial susceptibility testing were performed in the hospital microbiology laboratory according to the EUCAST clinical breakpoints applicable at the time of testing. Colistin susceptibility was tested by broth dilution method. The unit of analysis was the microbiological isolate rather than the individual patient, and repeated isolates from the same patient were included when obtained from separate clinical samples.

Statistical analysis of the collected data was performed using IBM SPSS Statistics, version 23.0. (IBM Corp., Armonk, NY, IBM Corp.), and Microsoft Excel (version 2606, Microsoft Corporation, Redmond, WA, USA). Since all analysed variables were categorical, the results of the categorical variables are presented as numbers (n) and percentages (%), and the Chi-squared test (*χ*2 test) or Fisher's exact test were used to test the significance of the differences. The level of statistical significance was set at *p* < 0.05, with a 95% confidence interval (CI).

## 3 Results

A total of 1,469 microbiological samples were retrieved from the hospital database. We immediately excluded 158 negative samples from analysis. Of the remaining 1,311 positive samples, we also excluded 198 with Candida species. A total of 1,113 microbiological isolates were included in this study: 238 microbiological samples from the multidisciplinary ICU (MICU) during the study period, from January 1 to December 31, 2017; 428 microbiological samples from the surgical ICU (SICU); and 447 microbiological samples from the nonsurgical ICU (NSICU) during the study period, from January 1 to December 31, 2024. The flowchart (Figure 1) shows the total number of analyzed samples and the distribution of isolated pathogens.

**Figure 1:**
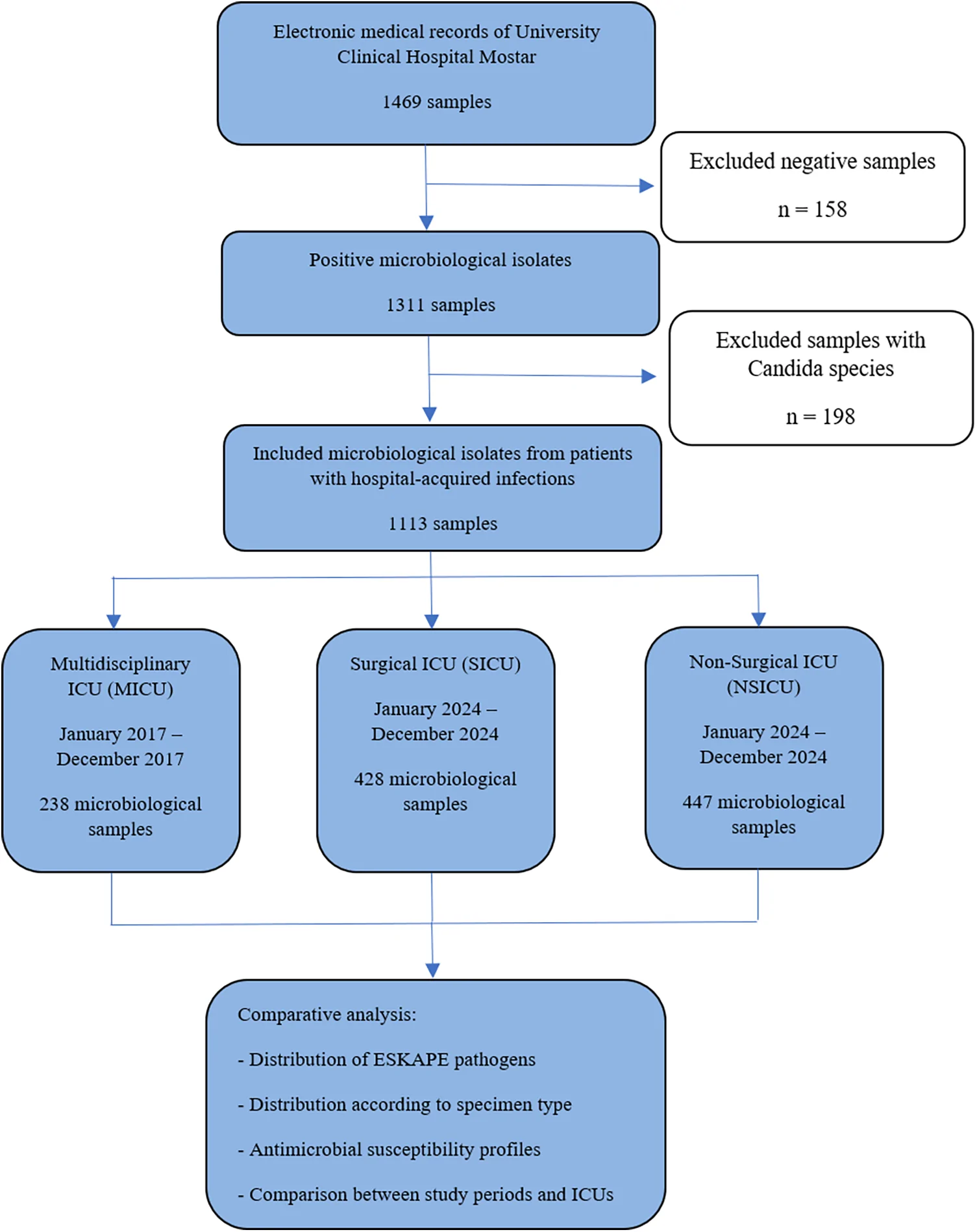
Flowchart onf samples enrollment.

The most common isolated pathogen in MICU and NSICU (Table 1) was *Acinetobacter baumannii* (31.5% and 30.4%, respectively)*,* which is significantly higher than the 21.7% isolated in SICU *(p* *=* 0.005, 95% CI and *p* *=* 0.003*,* 95% CI, respectively). *Klebsiella pneumoniae* was the most commonly isolated pathogen in the SICU (33.8%), which is significantly higher than the rates of 7.1% in the MICU (*p* < 0.001, 95% CI) and 22.6% in the NSICU (*p* < 0.001, 95% CI). *Pseudomonas aeruginosa* was present in 18.9% of isolates in the MICU, which is higher than 15.4% in the NSICU and significantly higher than 12.1% in the SICU (*p* = 0.017, 95% CI). *Enterococcus faecium* was also significantly higher in MICU (13.9%) than SICU (3.5%, *p* < 0.001, 95% CI) and NSICU (7.2%, *p* = 0.004, 95% CI). S*taphylococcus aureus* was isolated in about 1% at all ICUs, while non-ESKAPE pathogens were present in 22.8% - 28% of antimicrobial samples in all three ICUs. There was no statistically significant difference between them.

**Table 1:** Distribution of all Acquired samples.

All samples													
	Total number of samples	*Acinetobacter baumannii*		*Pseudomonas aeruginosa*		*Klebsiella pneumoniae*		*Enterococcus faecium*		*Staphylococcus aureus*		*Other pathogens*	
	*N*	*N*	%	*N*	%	*N*	%	*N*	%	*N*	%	*N*	%
MICU (2017)	238	75	31.5	45	18.9	17	7.1	33	13.9	4	1.7	64	26.9
SICU (2024)	428	93	21.7	52	12.1	145	33.8	15	3.5	4	0.9	119	28
p*		***p*** **=** **0.005**		***p*** **=** **0.017**		***p*** **<** **0.001**		***p*** **<** **0.001**		*p* = 0.36		*P* = 0.761	
NSICU (2024)	447	136	30.4	69	15.4	101	22.6	32	7.2	7	1.6	102	22.8
p**		*P* = 0.766		*p* = 0.241		***p*** **<** **0.001**		***P*** **=** **0.004**		*P* = 0.921		*P* = 0.233	
p***		***p*** **=** **0.003**		*p* = 0.156		***p*** **<** **0.001**		***P*** **=** **0.015**		*P* = 0.353		*P* = 0.077	

MICU: multidisciplinary ICU; SICU: surgical ICU; NSICU: non surgical ICU; N: number, %: percentage; p* - MICU vs. SICU; P** - MICU vs. NSICU; p*** - SICU vs. NSICU;/- no difference between tested samples.

The prevalence of antimicrobial samples, categorized by specimen type, is depicted in Table 2. In blood culture samples, non-ESKAPE pathogens were isolated at rates ranging from 36.7% to 47.3%. There was no statistical difference in their prevalence among ICUs. *A. baumannii* was isolated in approximately 25% of samples in the MICU and NSICU (25% and 24.6%, respectively), which was significantly higher than the 12.9% in the SICU (*p* = 0.024, 95% CI and *p* = 0.008, 95% CI, respectively). *K. pneumoniae* was the most common pathogen in blood cultures in SICU (32.8%), which was significantly higher than 13.3% in MICU (*p* = 0.003, 95% CI) and 15.8% in NSICU (*p* = 0.001, 95% CI). *P. aeruginosa* was present at a significantly lower rate at SICU (6.5%) than MICU (20%, *p* = 0.001, 95% CI).

**Table 2:** Distribution of ESKAPE pathogens by specimen type.

	MICU (2017)		SICU (2024)		P*	NSICU (2024)		P**	P***
	*N*	%	*N*	%		*N*	%		
Blood cultures									
*Acinetobacter baumannii*	15	25	26	12.9	***p*** **=** **0.024**	28	24.6	*p* = 0.953	***p*** **=** **0.008**
*Klebsiella pneumoniae*	8	13.3	66	32.8	***p*** **=** **0.003**	18	15.8	*p* = 0.661	***p*** **=** **0.001**
*Pseudomonas aeruginosa*	12	20	13	6.5	***p*** **=** **0.001**	14	12.3	*p* = 0.177	*p* = 0.078
*Enterococcus faecium*	3	5	5	2.5	*p* = 0.325	0	0	***p*** **=** **0.016**	*p* = 0.089
*Staphylococcus Aureus*	0	0	2	1	*p* = 0.437	0	0	/	*p* = 0.284
Other pathogens	22	36.7	89	44.3	*p* = 0.297	54	47.3	*p* = 0.181	*p* = 0.607
Total number of samples	60	100	201	100		114	100		
Bronchial aspirate									
*Acinetobacter baumannii*	53	44.5	61	34.8	*p* = 0.094	89	43	*p* = 0.792	*p* = 0.102
*Klebsiella pneumoniae*	6	5	49	28	***p*** **<** **0.001**	28	13.5	***p*** **=** **0.016**	***p*** **<** **0.001**
*Pseudomonas aeruginosa*	29	24.4	39	22.3	*p* = 0.675	49	23.6	*p* = 0.87	*p* = 0.763
*Enterococcus faecium*	3	2.5	1	0.6	P*p* = 0.168	2	1	*p* = 0.291	*p* = 0.665
*Staphylococcus Aureus*	4	3.4	2	1.2	Pp = 0.195	7	3.4	/	*p* = 0.161
Other pathogens	24	20.2	23	13.1	*p* = 0.103	32	15.5	*p* = 0.279	*p* = 0.506
Total number of samples	119	100	175	100		207	100		
Urine cultures									
*Acinetobacter baumannii*	7	11.9	6	11.5	*p* = 0.948	19	15.1	*p* = 0.56	*p* = 0.53
*Klebsiella pneumoniae*	3	5.1	30	57.7	***p*** **<** **0.001**	55	43.6	***p*** **<** **0.001**	*p* = 0.087
*Pseudomonas aeruginosa*	4	6.8	0	0	*p* = 0.056	6	4.8	*p* = 0.577	*p* = 0.109
*Enterococcus faecium*	27	45.7	9	17.3	***p*** **=** **0.001**	30	23.8	***p*** **=** **0.002**	*p* = 0.341
*Staphylococcus Aureus*	0	0	0	0		0	0		
Other pathogens	18	30.5	7	13.5	***p*** **=** **0.033**	16	12.7	***p*** **=** **0.003**	*p* = 0.885
Total number of samples	59	100	52	100		126	100		

MICU: multidisciplinary ICU; SICU: surgical ICU; NSICU: non surgical ICU; N: number, %: percentage; p* - MICU vs. SICU; P** - MICU vs. NSICU; p*** - SICU vs. NSICU;/- no difference between tested samples.

*A. baumannii* was the most prevalent pathogen in bronchial aspirate samples across all three ICUs, ranging from 34.8% to 44.5%. While *K. pneumoniae* was relatively uncommon in MICU (5%), its prevalence was significantly higher in NSICU (13.5%, *p* = 0.016, 95% CI) and even higher in SICU (28%, *p* < 0.001, 95% CI). The prevalence of *P. aeruginosa* was approximately 23% in all ICUs, while the prevalence of *S. aureus* and *E. faecium* was less than 4%.

*E. faecium* was the most common pathogen isolated from urine samples in MICU (45.7%), which is significantly higher than 17.3% in SICU (*p* = 0.001, 95% CI) and 23.8% in NSICU (*p* = 0.002, 95% CI). *K. pneumoniae* was present in 57.7% of samples in SICU and 43.6% of samples in NSICU, which is higher than 5.1% in MICU (*p* < 0.001, 95% CI). *A. baumannii* was present in 11.5% - 15.1% of tested samples, while *P. aeruginosa* was isolated in less than 7% of tested samples across all ICUs.

*A. baumannii* has shown a significant increase in resistance to the tested antibiotics (Table 3) from 2017 to 2024. While partially sensitive to many antibiotics and almost non-resistant to ampicillin/sulbactam and colistin in 2017 (MICU), *Acinetobacter* has shown partial resistance to colistin and tigecycline, with over 75% resistance to ampicillin/sulbactam and full resistance to all other antibiotics in 2024.

**Table 3:** Antibiotic resistance of *Acinetobacter baumanii.*

Acinetobacter baumannii												
	MICU (2017)			SICU (2024)			P*	NSICU (2024)			P**	P***
	Total (*N*)	Resistance		Total (*N*)	Resistance			Total (*N*)	Resistance			
		*N*	%		*N*	%			*N*	%		
amoxicillin/clavulanate	75	75	100	93	93	100	**/**	136	136	100	/	**/**
amikacin	75	50	66.6	93	93	100	***p*** **<** **0.001**	136	136	100	***p*** **<** **0.001**	/
ceftazidime	75	75	100	93	93	100	/	136	136	100	/	/
ciprofloxacin	75	75	100	93	93	100	/	136	136	100	/	/
ceftriaxone	75	74	98.6	93	93	100	*p* = 0.253	136	136	100	*p* = 0.253	/
colistin	75	0	0	74	0	0	**/**	136	3	2.2	*p* = 0.196	*p* = 0.196
cefuroxime	75	75	100	93	93	100	/	136	136	100	/	/
gentamicin	75	72	96	93	93	100	*p* = 0.052	136	136	100	***p*** **=** **0.019**	**/**
imipenem	75	71	94.6	93	93	100	***p*** **=** **0.023**	136	136	100	***p*** **=** **0.006**	/
ampicillin/sulbactam	75	8	10.6	90	71	78.8	***p*** **<** **0.001**	129	99	76.7	***p*** **<** **0.001**	*p* = 0.714
piperacillin/tazobactam	75	72	96	93	93	100	*p* = 0.052	136	136	100	***p*** **=** **0.019**	/
ceftazidime/avibactam				84	83	100		96	96	100		/
trimethoprim/sulfamethoxazole	75	62	82.6	93	93	100	***p*** **<** **0.001**	136	136	100	***p*** **<** **0.001**	/
meropenem	75	75	100	93	93	100	**/**	136	136	100	**/**	/
levofloxacin	75	75	100					5	5	100	**/**	
fosfomycin	75	60	80	9	9	100	*p* = 0.141	18	18	100	***p*** **=** **0.039**	/
tigecycline				93	7	7.5		136	1	0.7		***p*** **=** **0.005**
nitrofurantoin	75	75	100	6	6	100	/	19	19	100	/	/

MICU: multidisciplinary ICU; SICU: surgical ICU; NSICU: non surgical ICU; N: number, %; percentage; p* - MICU vs. SICU; P** - MICU vs. NSICU; p*** - SICU vs. NSICU;/- no difference between tested samples.

Antibiotic resistance of *K. pneumoniae* has shown a similar trend of increased antibiotic resistance in the period 2017–2024 (Table 4). Interestingly, while being more resistant to amoxicillin/clavulanate, amikacin, and gentamicin than in 2017 (MICU), Klebsiella has shown less resistance to those antibiotics in NSICU than in SICU (2024).

**Table 4:** Antibiotic resistance of *Klebsiella pneumoniae.*

Klebsiella pneumoniae												
	MICU (2017)			SICU (2024)			P*	NSICU (2024)			P**	P***
	Total (*N*)	Resistance		Total (*N*)	Resistance			Total (*N*)	Resistance			
		*N*	%		*N*	%			*N*	%		
amoxicillin/clavulanate	17	14	82.3	132	129	97.7	***p*** **=** **0.002**	100	90	90	*p* = 0.352	***p*** **=** **0.011**
amikacin	17	1	5.8	136	109	80.1	***p*** **<** **0.001**	101	44	43.5	***p*** **=** **0.003**	***p*** **<** **0.001**
ceftazidime	17	17	100	144	135	93.7	*p* = 0.288	101	88	87.1	*p* = 0.118	*p* = 0.076
ciprofloxacin	17	14	82.3	145	134	92.4	*p* = 0.162	101	88	87.1	*p* = 0.594	*p* = 0.169
ceftriaxone	17	17	100	145	136	93.8	*p* = 0.292	101	88	87.1	*p* = 0.118	*p* = 0.07
colistin	17	0	0	22	5	22.7	***p*** **=** **0.037**	32	13	40.6	***p*** **=** **0.002**	*p* = 0.174
cefuroxime	17	15	88.2	145	136	93.8	*p* = 0.386	94	81	92.3	*p* = 0.575	*p* = 0.653
gentamicin	17	17	100	145	132	91	*p* = 0.198	101	78	77.2	***p*** **=** **0.028**	***p*** **=** **0.002**
imipenem	17	0	0	143	108	75.5	***p*** **<** **0.001**	99	73	73.7	***p*** **<** **0.001**	*p* = 0.751
ampicillin/sulbactam	17	17	100	141	135	95.7	*p* = 0.384	82	81	98.8	*p* = 0.651	*p* = 0.203
piperacillin/tazobactam	17	6	35.3	137	108	78.8	***p*** **<** **0.001**	90	75	83.3	***p*** **<** **0.001**	*p* = 0.51
ceftazidime/avibactam				136	57	41.9		84	33	39.3		*p* = 0.703
trimethoprim/sulfamethoxazole	17	8	47	145	134	92.4	***p*** **<** **0.001**	95	84	88.4	***p*** **<** **0.001**	*p* = 0.295
meropenem	17	0	0	143	108	75.5	***p*** **<** **0.001**	98	72	73.5	***p*** **<** **0.001**	*p* = 0.726
levofloxacin	17	0	0					3	2	66.6	***p*** **=** **0.043**	
fosfomycin	17	0	0	40	15	37.5	***p*** **=** **0.003**	37	21	56.7	***p*** **<** **0.001**	*p* = 0.093
tigecycline				109	0	0		95	2	2.1		*p* = 0.129
amoxicillin	17	17	100					20	20	100	/	
nitrofurantoin				30	30	100		55	55	100		/

MICU: multidisciplinary ICU; SICU: surgical ICU; NSICU: non surgical ICU; N: number, %: percentage; p* - MICU vs. SICU; P** - MICU vs. NSICU; p*** - SICU vs. NSICU;/- no difference between tested samples.

The antibiotic resistance of *P. aeruginosa*, as presented in Table 5, exhibits mixed trends. This pathogen has become significantly more sensitive to amikacin (NSICU and SICU) and ciprofloxacin (SICU) than in 2017. Resistance to piperacillin/tazobactam has increased in the SICU and NSICU, whereas resistance to ceftazidime and cefepime has increased in the SICU compared to the MICU and NSICU.

**Table 5:** Antibiotic resistance of *Psudomonas aeruginosa.*

Pseudomonas aeruginosa												
	MICU (2017)			SICU (2024)			P*	NSICU (2024)			P**	P***
	Total (*N*)	Resistance		Total (*N*)	Resistance			Total (*N*)	Resistance			
		*N*	%		*N*	%			*N*	%		
amikacin	45	9	20	52	1	1.9	***p*** **=** **0.003**	69	0	0	***p*** **<** **0.001**	*p* = 0.252
ceftazidime	45	3	6.7	50	12	24	***p*** **=** **0.021**	69	2	2.9	*p* = 0.335	***p*** **<** **0.001**
ciprofloxacin	45	22	48.9	51	12	23.5	***p*** **=** **0.009**	66	25	37.9	*p* = 0.251	*p* = 0.098
colistin	45	0	0	17	0	0	**/**	69	0	0	**/**	/
cefepime	45	0	0	38	5	13.2	***p*** **=** **0.012**	56	1	1.8	*p* = 0.368	***p*** **=** **0.027**
gentamicin	45	18	40					66	33	50	*p* = 0.301	
imipenem	45	10	22.2	39	13	33.3	*p* = 0.257	65	31	47.7	***p*** **=** **0.006**	*p* = 0.152
piperacillin/tazobactam	45	0	0	52	10	19.2	***p*** **=** **0.002**	61	10	16.4	***p*** **=** **0.004**	*p* = 0.698
ceftazidime/avibactam				8	0	0		15	0	0		/
meropenem	45	17	37.8	42	16	38	*p* = 0.984	64	33	51.5	*p* = 0.159	*p* = 0.174
levofloxacin	45	22	48.9					16	8	50	*p* = 0.94	
tobramycin	45	12	26.7	43	10	23.2	*p* = 0.706	55	18	32.7	*p* = 0.516	*p* = 0.303

MICU: multidisciplinary ICU; SICU: surgical ICU; NSICU: non surgical ICU; N: number, %: percentage; p* - MICU vs. SICU; P** - MICU vs. NSICU; p*** - SICU vs. NSICU;/- no difference between tested samples.

The resistance of *E. faecium* to ciprofloxacin has significantly increased in 2024 (SICU and NSICU) compared to 2017 (Table 6). Resistance to nitrofurantoin has also increased in NSICU compared to MICU, while resistance to ampicillin was reported to be greater in NSICU than in both MICU and SICU.

**Table 6:** Antibiotic resistance of *Enterococcus faecium.*

Enterococcus faecium												
	MICU (2017)			SICU (2024)			P*	NSICU (2024)			P**	P***
	Total (*N*)	Resistance		Total (*N*)	Resistance			Total (*N*)	Resistance			
		*N*	%		*N*	%			*N*	%		
amoxicillin/clavulanate	33	12	36.4	15	4	26.7	*p* = 0.513	32	16	50	*p* = 0.272	*p* = 0.136
vancomycin	33	7	21.2	15	0	0	*p* = 0.056	32	3	9.4	*p* = 0.19	*p* = 0.224
ciprofloxacin	33	20	60.6	15	14	93.3	***p*** **=** **0.022**	29	27	93.1	***p*** **=** **0.003**	*p* = 0.98
Linezolid	33	0	0					21	0	0	/	
teicoplanin	33	8	24.2	15	0	0	***p*** **=** **0.038**	32	4	12.5	*p* = 0.227	*p* = 0.156
gentamicin	33	22	66.7	15	14	93.3	*p* = 0.05	32	25	78.1	*p* = 0.308	*p* = 0.201
ampicillin	33	8	24.2	12	2	16.7	*p* = 0.596	32	20	62.5	***p*** **=** **0.002**	***p*** **=** **0.007**
amoxicillin	33	13	39.4	12	4	33.3	*p* = 0.712	32	20	62.5	*p* = 0.064	*p* = 0.086
nitrofurantoin	33	10	30.3	9	4	44.4	*p* = 0.431	30	20	66.7	***p*** **=** **0.004**	= 0.233

MICU: multidisciplinary ICU; SICU: surgical ICU; NSICU: non surgical ICU; N: number, %: percentage; p* - MICU vs. SICU; P** - MICU vs. NSICU; p*** - SICU vs. NSICU;/- no difference between tested samples.

## 4 Discussion

The emergence of antibiotic resistance in ESKAPE pathogens causing hospital-acquired infections (HAI) is a global threat to worldwide human health, as it causes treatment failure and the reduction of available treatment options for severe infections, thus increasing mortality rates (8). Although significant advances in intensive care have been made, HAI remain a potentially modifiable source of morbidity and mortality. The prevalence of HAI remains significantly higher in ICU patients than among non-ICU patients (9).

The present study demonstrates that ESKAPE pathogens continue to dominate the microbiological landscape of intensive care units in our institution, accounting for more than 70% of microbiologically confirmed isolates during both study periods. More importantly, comparison of data collected seven years apart revealed substantial changes in pathogen distribution accompanied by a marked increase in antimicrobial resistance among the most clinically relevant Gram-negative pathogens.

To our knowledge, this is the first study from Bosnia and Herzegovina to compare changes in the epidemiology of ESKAPE pathogens and their antimicrobial resistance across different time periods and ICU settings. ESKAPE pathogens were the cause of over 70% of HAI in all three ICUs during two study periods (2017 and 2024). Although some authors report an increase in the prevalence of these bacteria during the observed study period (10), in our case, the overall proportion of ESKAPE pathogens remained relatively stable at 70%, similar to that reported in the ICU in Romania (11) while the distribution of individual microorganisms changed considerably. This finding suggests that local epidemiology should not be interpreted only through the overall prevalence of resistant pathogens but also through changes in the relative predominance of individual bacterial species.

One of the most interesting findings of this study was the epidemiological shift observed between *A. baumannii* and *K. pneumoniae*. Although *A. baumannii* remained the predominant pathogen in the MICU in 2017 and in the NSICU in 2024, *K. pneumoniae* became the leading pathogen in the SICU (2024).

A similar predominance of *A. baumannii* in intensive care settings has been described previously (12). However, a significant increase in *K. pneumoniae* isolates, especially in SICU, is in line with the epidemiology in other ICUs (11, 13) and can be explained by differences in patient population rather than a simple increase in pathogen prevalence. Surgical patients are more frequently exposed to invasive procedures, prolonged mechanical ventilation, urinary catheterization, and broad-spectrum antibiotic therapy. Along with a history of solid tumors and previous invasive procedures, these are all recognized risk factors for colonization and infection caused by *K. pneumoniae* (14). Furthermore, the reorganization of our intensive care service into separate surgical and non-surgical units likely contributed to differences in microbial isolates between these patient populations.

Regarding specimen type, ESKAPE pathogens in 2024 were mainly isolated from urine cultures, followed by bronchial aspirate and blood culture samples, respectively. The highest distribution of ESKAPE pathogens in urine samples was also reported by similar studies that explored the distribution of ESKAPE pathogens in hospital wards (10, 13, 15). These results indicate a change in trend compared to year 2017, when most of the ESKAPE pathogens were isolated from bronchial aspirate samples, followed by urine and blood culture samples, respectively.

Resistance trends for *A. baumannii, K. pneumoniae, P. aeruginosa, and E. faecium* (Tables 3–6) *varied significantly between the* tested ICUs. While the prevailing trend, in the period 2017–2024, is increased resistance to tested antibiotics, some of the selected pathogens showed a decreasing resistance trend.

*A. baumannii* infection rates are considered to be relatively low compared to those of other ESKAPE pathogens, but around 45% of all global *A. baumannii* isolates are considered MDR (16), with rates from 60% in the United States, Latin America, and the Middle East to over 90% in Turkey and Greece (17)*.* The rapid development of antimicrobial resistance is one of the main features of *A. baumannii* physiology, and with the emergence of pandrug-resistant isolates, last-resort carbapenem and polymyxin class antibiotics are often rendered ineffective (17).

In our study, *A. baumannii* showed a significant increase in antibiotic resistance in the investigated 7-year period. Even in MICU (2017), *Acinetobacter* was a multidrug-resistant (MDR) pathogen that showed no resistance only to colistin and minimal resistance to ampicillin/sulbactam. Apart from amikacin (66.6% resistance), *Acinetobacter* showed complete or near-maximal resistance to all other major antimicrobial groups (*β*-lactams, aminoglycosides, fluoroquinolones, carbapenems, etc). Such results are similar to those reported by other authors (18), although there are still reports from other ICUs where this pathogen didn't express complete resistance to named antimicrobial groups (19). These high resistance rates from 2017 even increased in both ICUs in 2024, a trend also reported in other studies (9). Resistance to ampicillin/sulbactam increased significantly during the tested period, reaching almost 80% in both SICU and NSICU. Apart from colistin and the newly acquired tigecycline, *A. baumannii* became fully resistant to all other tested antibiotics, leaving these two as the only viable therapeutic options; even signs of early resistance to these two agents became apparent.

Cephalosporins and carbapenems have for a long time been a mainstay of treatment for serious infections caused by *K. pneumoniae*, but their efficacy has been compromised by the widespread acquisition of genes encoding extended-spectrum-lactamases (ESBLs) and carbapenemases, enzymes which mediate resistance to these critical drugs (17). In our study, *K. pneumoniae* exhibited a trend of increasing antimicrobial resistance similar to that of *A. baumannii.* While being fully susceptible to carbapenems in 2017, carbapenem-resistant strains were present in around 75% of isolated samples in 2024. Resistance also increased substantially to aminoglycosides, fluoroquinolones and *β*-lactam/*β*-lactamase inhibitor combinations, whereas susceptibility to tigecycline and ceftazidime-avibactam remained relatively preserved. These results are similar to most of those reported in other studies (9, 20), although some authors reported lower resistance rates (8). We also observed that resistance patterns differed between the surgical and non-surgical ICUs, which could suggest that local antimicrobial prescribing practices and patient characteristics may influence resistance development even within the same institution.

*P. aeruginosa* is an opportunistic human pathogen reported to be responsible for 10% of nosocomial infections. However, there are also increasing reports of *P. aeruginosa* as a cause of community-acquired infections (17). It is intrinsically resistant to multiple classes of antimicrobial agents due to multifactorial, intrinsic, and acquired mechanisms, which include the acquisition of mobile genetic elements, such as integrons and plasmids, or mutations in chromosomal genes (21). The global rate of *P. aeruginosa* antibiotic resistance is variable. However, surveillance data from the United States report a downward trend of resistance (17). Our research showed a mixed trend of *P. aeruginosa* resistance. Resistance rates to carbapenems, especially imipenem, increased, reaching up to 50%, similar to reports from other authors (15, 21).

The relatively low prevalence of *S. aureus* and the limited number of *E. faecium* isolates prevented detailed epidemiological conclusions regarding these organisms. Nevertheless, the observed increase in ciprofloxacin and ampicillin resistance among *E. faecium* isolates is consistent with the continuing global emergence of multidrug-resistant enterococci in healthcare settings (22).

The principal strengths of this study include the comparison of microbiological data over a seven-year interval and the detailed analysis of antimicrobial resistance profiles of the most clinically important ESKAPE pathogens from different intensive care settings within a tertiary hospital. However, several limitations should be acknowledged. This was a retrospective single-center study, limiting the generalisability of our findings. Furthermore, the microbiological isolate, rather than the individual patient, was the unit of analysis, and we included repeated isolates from the same patient when obtained from separate clinical samples. The reorganization of the intensive care service between study periods may also have influenced pathogen distribution independently of temporal changes.

In conclusion, this study demonstrates that although the overall proportion of ESKAPE pathogens remained relatively stable over seven years, important changes occurred in the distribution of individual pathogens together with a substantial increase in antimicrobial resistance, particularly among *Acinetobacter baumannii* and *Klebsiella pneumoniae*. The observed changes in pathogen distribution and antimicrobial susceptibility emphasize that empirical antibiotic protocols should be based on continuously updated local surveillance data rather than historical resistance patterns or national estimates. Regular institutional surveillance enables optimization of empirical antimicrobial therapy, supports antimicrobial stewardship programs, and provides an evidence base for targeted infection prevention within intensive care units.

## Data Availability

The raw data supporting the conclusions of this article will be made available by the authors, without undue reservation.
